# KLF4 down‐regulation resulting from TLR4 promotion of ERK1/2 phosphorylation underpins inflammatory response in sepsis

**DOI:** 10.1111/jcmm.16082

**Published:** 2020-12-27

**Authors:** Chunwen Li, Lei Yu, Chao Mai, Tianyi Mu, Yong Zeng

**Affiliations:** ^1^ Emergency Department The Second Affiliated Hospital of Chongqing Medical University Chongqing China; ^2^ Emergency Department Affiliated Hospital of North Sichuan Medical College Nanchong China

**Keywords:** extracellular regulated kinase 1/2, inflammation, Integrin Alpha 2B, Krüppel‐like factor 4, sepsis, Toll‐like receptor 4

## Abstract

Sepsis is a systemic inflammatory response to invading pathogens, leading to high mortality rates in intensive care units worldwide. Krüppel‐like factor 4 (KLF4) is an important anti‐inflammatory transcription factor. In this study, we investigate the anti‐inflammatory role of KLF4 in caecal ligation and puncture (CLP)‐induced septic mice and lipopolysaccharide (LPS)‐induced RAW264.7 cells and its potential mechanism. We found that KLF4 was down‐regulated in CLP‐induced septic mice and in LPS‐induced RAW264.7 cells, and that its overexpression led to increased survival rates of septic mice along with inhibited inflammatory response in vivo and in vitro. ITGA2B was up‐regulated in the setting of sepsis and was inhibited by KLF4 overexpression. ITGA2B knock‐down mimicked the effects of KLF4 overexpression on septic mice and LPS‐induced RAW264.7 cells. TLR4 promoted the phosphorylation of ERK1/2 and then up‐regulated the ubiquitination and the degradation of KLF4, thereby elevating the expression of ITGA2B. Moreover, TLR4 knock‐down or treatment with PD98059 (a MEK inhibitor) inhibited inflammatory response in the setting of sepsis in vivo and in vitro. Furthermore, this effect of PD98059 treatment was lost upon KLF4 knock‐down. Collectively, these results explain the down‐regulation of KLF4 in sepsis, namely via TLR4 promotion of ERK1/2 phosphorylation, and identify ITGA2B as the downstream gene of KLF4, thus highlighting the anti‐inflammatory role of KLF4 in sepsis.

## 1 INTRODUCTION

Sepsis and septic shock accompanied by following multi‐organ failure are considered are among the principle reasons for deaths of adults in intensive care units.^1^ Based on data from high‐income nations, it is tentatively extrapolated that 31.5 million cases of sepsis are diagnosed annually, of which 19.4 million are severe cases leading to death in as many as 5.3 million individuals per year.^2^ Despite advancements of management achieved in the past few years, patients who have survived sepsis often suffer from persistent physical, physiologic and psychological sequelae, including neurocognitive and functional decline, and have an increased overall risk of mortality,^3^, ^4^ highlighting the need to identify novel therapeutic targets and strategies against sepsis.

Given the involvement of innate and adaptive immune responses in sepsis, investigations focusing on regulatory mechanism underlying immune responses are expected to yield better understanding and improvement of long‐term clinical outcomes of patients with sepsis.^5^ Krüppel‐like factor 4 (KLF4), which is as an evolutionarily conserved transcription factor containing zinc fingers, is known for its mediatory role in various cellular processes, including proliferation, differentiation and inflammation.^6^ In addition, KLF4 has been reported to be expressed in overt inflammatory conditions.^7^ Upon exposure to lipopolysaccharide (LPS), RAW264.7 cells show a suppression of KLF4 expression along with enhanced release of pro‐inflammatory cytokines,^8^ implying that KLF4 may be engaged in the immune response in sepsis. RNA‐sequencing analysis performed by Xiong et al^9^ revealed downstream genes targeted by KLF4, including the ITGA2B gene. Its protein transcript, integrin Alpha 2B (ITGA2B), was increased in circulating platelets and associated with higher mortality of patients and mice with sepsis.^10^ Accordingly, ITGA2B merits study as the downstream of KLF4 in sepsis. Besides, phosphorylation of KLF4 has been indicated to be mediated by extracellular regulated kinase 1/2 (ERK1/2) in embryonic stem cells through ubiquitination and degradation.^11^ The activation of ERK1/2 has been elucidated to participate in the regulatory role of insulin‐like growth factor bind protein 7 in LPS‐induced HK‐2 cells and in the mouse sepsis model entailing caecal ligation and puncture (CLP).^12^ Furthermore, the phosphorylation of ERK1/2 can be diminished by blocking toll‐like receptor 4 (TLR4) in myeloid‐derived suppressor cells, which are enriched in chronic inflammatory conditions, thus implicating ERK1/2 phosphorylation in disorders related to inflammation.^13^ Also, activation of TLR4 has been detected as part of the inflammatory response in patients with liver injury induced by sepsis.^14^ Herein, we investigate the hypothesis that the TLR4/ERK1/2/KLF4/ITGA2B axis mediates the inflammatory response of sepsis. To test this hypothesis, we established CLP‐induced septic mice and LPS‐induced RAW264.7 cells and characterized the role of the TLR4/ERK1/2/KLF4/ITGA2B axis in the setting of sepsis in vivo and in vitro.

## 2 MATERIALS AND METHODS

### 2.1 Ethics statement

The study was approved by the ethics committee of experimental animal centre of Chongqing Medical University. All experiments were in accordance with the Declaration of Helsinki.

### 2.2 Establishment of sepsis mouse model

In total, 240 male C57BL/6 mice (age: 6‐8 weeks, weight: 20‐25 g) were fasted for 12 hours and then anaesthetized through intraperitoneal injection of 2.5% pentobarbital sodium (2 mL/kg) prior to surgery. In this group, 20 mice served as controls with surgical laparotomy to isolate the distal pole of the caecum and mesentery, followed by abdominal closure. The remaining 220 mice were subjected to CLP surgery for induction of high‐grade sepsis. In brief, the abdomen was conventionally disinfected and opened by creating a 2‐cm incision in the middle to expose the caecum. Next, the distal pole of the caecum was separated from the mesentery to avoid damaging the mesenteric vessels. Subsequently, sterile No.4 thread was ligated at 3/4 of the way from the distal pole of the caecum. Then, the caecum was perforated by single through‐and‐through puncture at the midway between the ligation and the tip of the caecum using a sterile No.7 pipette. Finally, the abdomen was closed with layer‐by‐layer suturing. Lentiviruses harboring KLF4 overexpression plasmid (oe‐KLF4), ITGA2B overexpression plasmid (oe‐ITGA2B), short hairpin RNA (shRNA) against ITGA2B (sh‐ITGA2B), shRNA against TLR4 (sh‐TLR4), shRNA against KLF4 (sh‐KLF4), or the corresponding negative controls (NCs) (oe‐NC and sh‐NC) (1.5 × 10^9^ TU) were injected into the CLP‐induced septic mice via a tail vein at 4 hours after the surgery.^15^, ^16^ Dimethyl sulphoxide (DMSO) or PD98059 (ERK1/2 inhibitor; Sigma‐Aldrich) dissolved in DMSO was intraperitoneally injected into the CLP‐induced septic mice (10 mg/kg), while control mice were treated with administration of DMSO alone. At 48 hours after surgery, the mice were killed with sodium pentobarbital overdose (100 mg/kg; P3761, Sigma‐Aldrich).

### 2.3 Establishment of sepsis cell model

Murine RAW264.7 macrophages (#TIB‐71; ATCC) were cultured in DMEM (Gibco) containing 10% foetal bovine serum, 100 mg/mL streptomycin and 100 U/mL penicillin. When the cell confluence reached 60%‐80%, RAW264.7 cells were treated with oe‐NC, sh‐NC, sh‐ITGA2B, oe‐KLF4, oe‐ITGA2B or sh‐TLR4 (all Gene Pharma Co., Ltd.) using Lipofectamine 3000 (#L3000008; Invitrogen) or treated with DMSO or PD98059 (MEK inhibitor; Cell Signaling Technology) diluted in DMSO (20 μmol/L). All plasmids were purchased from Gene Pharma Co., Ltd. 48 hours later, RAW264.7 cells were stimulated by treatment with 1 μg/mL LPS (Escherichia coli 055: B5; Sigma‐Aldrich) for 48 hours.^17^

### 2.4 Real‐time quantitative polymerase chain reaction

Extraction of total RNA was made by using TRIZOL (Invitrogen). The primer sequences of TLR4, KLF4 and ITGA2B, which were designed and constructed by Invitrogen, are shown in Table 1. The obtained total RNA was reversely transcribed to cDNA following the instructions of the High‐Capacity cDNA Reverse Transcription Kit (4368813; Thermo Fisher Scientific). Real‐time quantitative polymerase chain reaction (RT‐qPCR) was carried out using SYBR^®^Premix Ex Taq™ (Tli RNaseH Plus) kit (RR820A, TaKaRa) on an ABI7500 qPCR instrument (Thermo Fisher Scientific). The expression of the target gene relative to GAPDH was analysed by 2 ^–ΔΔ^ *^C^* ^t^ method.^18^

**Table 1:** Primer sequences for RT‐qPCR

Genes	Primer sequences (5′‐3′)
TLR4	F: 5′‐ACTCTGATCATGGCACTGTTCTT‐3′
	R: 5′‐GCTCAGATCTATGTTGGTTGA‐3′
KLF4	F: 5′‐TTGCGGTAGTGCCTGGTCAGTT‐3′
	R: 5′‐TGATGTCCTTCACGCTCAGG‐3′
ITGA2B	F: 5′‐AAGCTCTGAGCACACCCACT‐3′
	R: 5′‐CTCAGCCCTTCACTCTGACC‐3′
GAPDH	F: 5′‐AGGTCGGTGTGAACGGATTTG‐3′
	R: 5′‐TGTAGACCATGTAGTTGAGGTCA‐3′

Abbreviations: F, forward; GAPDH, glyceraldehyde‐3‐phosphate dehydrogenase; ITGA2B, Integrin Alpha 2B; KLF4, Krüppel‐like factor 4; R, reverse; RT‐qPCR, reverse transcription‐quantitative polymerase chain reaction; TLR4, toll‐like receptor 4.

### 2.5 Western blot analysis

Total protein was extracted from tissues or RAW264.7 cells using Radio Immunoprecipitation Assay (RIPA) Lysis Buffer (R0010; Solarbio). After SDS‐PAGE separation and membrane transfer, immunoblots were obtained by using the following primary rabbit antibodies: GAPDH (#5174, 1:1000, Rabbit, Cell Signaling Technology), TLR4 (ab13556, 1:500; Abcam Inc.), phosphorylated‐ERK1/2 (p‐ERK1/2) (ab17942, 1:1000; Abcam), ERK1/2 (ab17942, 1:1000; Abcam), KLF4 (ab129473, 1:1000; Abcam) and ITGA2B (ab134131, 1:2000; Abcam). The immunoblots were visualized with secondary goat anti‐rabbit antibody to immunoglobulin G (ab150077, 1:1000; Abcam) and enhanced chemiluminescence, and analysed by Image J software. The relative expression of protein was expressed as the ratio of grey value of the target band to that of internal reference (GAPDH).

### 2.6 Protein stability assay

To detect the degradation of KLF4, tunicamycin (654380; Sigma‐Aldrich) was applied to treat the RAW264.7 cells, followed by application of 20 μM cycloheximide (HY‐12320; MedChemExpress) to inhibit protein synthesis. At different time points (0, 1, 2, 3 and 4 hours), RAW264.7 cells were lysed with RIPA Lysis Buffer (P0013B; Beyotime Biotechnology Co., Ltd.) and then incubated with PNGase F (G5166‐50UN; Sigma‐Aldrich). Next, proteins were isolated by centrifugation at 6037 *g*. The effects of PNGase F on KLF4 protein expression were explored by Western blot analysis, and the KLF4 degradation curve was plotted.

### 2.7 Immunoprecipitation

After 48 hours of transfection, cells were washed by pre‐cooled phosphate buffer saline (PBS) and incubated with immunoprecipitation (IP) lysis. After lysis on ice for 30 minutes, the supernatant was collected after centrifugation at 7446 *g* and 4°C for 20 minutes. Next, the protein concentration was measured by BCA method. 1 mg protein was incubated with the corresponding primary antibody at 4°C overnight. The next day, the protein was incubated for another 2 hours after the addition of 20 μL Protein A + G beads. Elution was followed using IP lysis through centrifugation at 1258 *g* and 4°C for 5 minutes, 5 times in total. With 20 μL 2 × Loading buffer in each well, samples were denatured by placement in a bath at 100°C for 5 minutes. The IP sample was subjected to Western blot analysis using antibodies to KLF4 (ab129473, 1:1000, Rabbit; Abcam) and ubiquitin (ab7780, 1:1000; Abcam).

### 2.8 Bacterial load test

At 48 hours after the operation, orbital blood samples were collected. Then, peritoneal lavage fluid (PLF) was harvested using 3.5 mL sterile normal saline. After gradual dilution, the blood and PLF were applied on trypsin blood agar plates and cultured at 37°C. The number of bacterial colonies was counted 24 hours later.

### 2.9 Haematoxylin‐eosin staining

The freshly collected liver and lung tissues were fixed with 4% paraformaldehyde at room temperature for at least 16 hours, embedded with paraffin and sliced into 3‐µm sections. The sections were treated with xylene I for 20 minutes, xylene II for 20 minutes, absolute alcohol I for 15 minutes, absolute alcohol II for 5 minutes, and 75% alcohol for 5 minutes. The sections were then stained with haematoxylin for 3‐5 minutes, followed by differentiation. After being blued, sections were dehydrated by 85% and then 95% alcohol for 5 minutes each. The sections were then stained with eosin for 5 minutes, followed by clearing with absolute alcohol I, absolute alcohol II, absolute alcohol III, xylene I and xylene II (5 minutes each). The mounted sections were then observed using a microscope. Immunohistochemistry was scored as described in a previous study, where absent inflammation scored as 0 points, neutrophil infiltration as 1 point, oedema as 2 points, cellular disorder as 3 points and bleeding as 4 points.^19^

### 2.10 Enzyme‐linked immunosorbent assay

After the different treatments, RAW264.7 cells were cultured for 48 hours and the supernatant was collected, which was then subjected to centrifugation and cryopreservation at −20°C. Mouse whole blood was centrifuged at 2000 *g* for 20 minutes to harvest the serum, which was then cryopreserved at −20°C. The liver and lung tissues were isolated in the presence of liquid nitrogen and mixed with 0.5 mL normal saline, followed by centrifugation at 2516 *g* for 10 minutes to collect the supernatant. Finally, the expression of pro‐inflammatory factors including tumour necrosis factor‐α (TNF‐α) (MTA00B), interleukin‐6 (IL‐6) (MLB00C) and IL‐1β (M6000B) in the cell culture medium supernatant, mouse serum and the supernatant of liver and lung tissue homogenate was measured following instructions of Enzyme‐linked immunosorbent assay (ELISA) kits (R&D Systems Inc.).

### 2.11 Statistical analysis

Data analysis was performed using SPSS 21.0 software (IBM). A *P* value of <.05 was considered as statistically significant.

## 3 RESULTS

### 3.1 KLF4 overexpression protected mice against CLP‐induced sepsis

**Figure 1:**
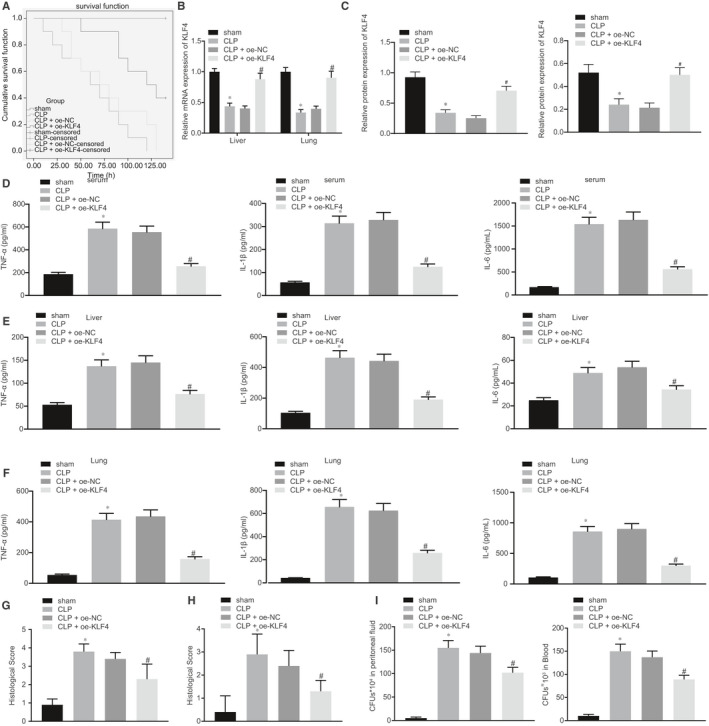
KLF4 overexpression protected mice against caecal ligation and puncture (CLP)‐induced sepsis, with KLF4 up‐regulation inhibiting sepsis in mice. A, The Kaplan‐Meyer survival plots of mice at 140 h after CLP surgery. B, The mRNA expression of KLF4 in mouse liver and lung tissues at 48 h after CLP surgery determined by RT‐qPCR. C, The protein expression of KLF4 in mouse liver and lung tissues at 48 h after CLP surgery determined by Western blot analysis, normalized to GAPDH. D, The serum levels of TNF‐α, IL‐1β and IL‐6 at 48 h after CLP surgery measured by ELISA. E, The levels of TNF‐α, IL‐1β and IL‐6 in mouse liver tissues at 48 h after CLP surgery evaluated by ELISA. F, The levels of TNF‐α, IL‐1β and IL‐6 in mouse lung tissues at 48 h after CLP surgery tested by ELISA. G, Morphological observation and score of liver tissues at 48 h after CLP surgery identified by Haematoxylin‐eosin (HE) staining (200×). H, Morphological observation and score of lung tissues at 48 h after CLP surgery identified by HE staining (200×). I, The number of bacterial colonies in mouse PLF and serum. *P < .05 vs the sham‐operated mice. ^#^ *P* < .05 vs the CLP‐induced septic mice treated with lentivirus harboring oe‐NC. N = 10. Data were expressed as mean ± standard deviation. Data between two groups were compared by independent‐sample *t* test. The experiments were conducted 3 times independently

The mouse model of sepsis was established to explore the functional role of KLF4. At the same time, lentiviruses harboring oe‐KLF4 were injected into the CLP‐induced septic mice. As shown in (Figure 1A), the all CLP‐induced septic mice eventually died. Comparatively, the overexpression of KLF4 improved their survival rate to more than 50%. In addition, results of RT‐qPCR and Western blot analysis revealed the levels of KLF4 were evidently diminished in liver and lung tissues of CLP‐induced septic mice, while that in the CLP‐induced septic mice treated with oe‐KLF4 were elevated (Figure 1B,C). Through ELISA analysis, the expression of pro‐inflammatory factors (TNF‐α, IL‐1β and IL‐6) in serum, liver tissues and lung tissues of CLP‐induced septic mice was significantly higher than that in CLP‐induced septic mice with up‐regulated expression of KLF4 (Figure 1D‐F). Pathological investigation after Haematoxylin‐eosin (HE) staining showed that CLP‐induced interstitial oedema, inflammatory cell infiltration and damage in the liver and lung tissues, all of which were effectively alleviated by overexpressed KLF4 (Figure 1G,H). Also, the number of bacterial colonies in the ascitic fluid and serum in CLP‐induced septic mice was less in the mice with KLF4 overexpression (Figure 1I). Taken together, KLF4 was expressed at a low level in mice with sepsis, but elevating KLF4 expression brought considerable alleviation of sepsis.

### 3.2 KLF4 overexpression protected mice against CLP‐induced sepsis by down‐regulating ITGA2B

**Figure 2:**
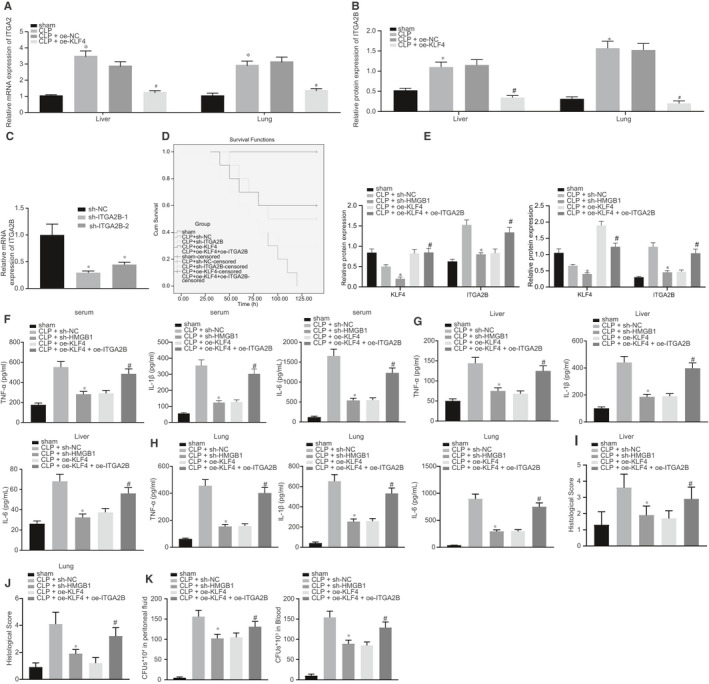
KLF4 overexpression protected mice against caecal ligation and puncture (CLP)‐induced sepsis by down‐regulating ITGA2B. A, The mRNA expression of ITGA2B in mouse liver and lung tissues at 48 h after CLP surgery determined by RT‐qPCR. B, The protein expression of ITGA2B in mouse liver and lung tissues at 48 h after CLP normalized to GAPDH detected by Western blot analysis, normalized to GAPDH. C, The silencing efficiency of ITGA2B in RAW264.7 cells measured by RT‐qPCR. D, The survival rate of mice at 140 h after CLP surgery. E, The protein expression of KLF4 and ITGA2B in mouse liver and lung tissues at 48 h after CLP surgery examined by Western blot analysis, normalized to GAPDH. F, The serum levels of TNF‐α, IL‐1β and IL‐6 at 48 h after CLP surgery determined by ELISA. G, The levels of TNF‐α, IL‐1β and IL‐6 in mouse liver tissues at 48 h after CLP surgery evaluated by ELISA. H, The levels of TNF‐α, IL‐1β and IL‐6 in mouse lung tissues at 48 h after CLP surgery detected by ELISA. I, Morphological observation and score of liver tissues at 48 h after CLP surgery identified by Haematoxylin‐eosin (HE) staining (200×). J, Morphological observation and score of lung tissues at 48 h after CLP surgery identified by HE staining (200×). K, The number of bacterial colonies in mouse PLF and serum. In panels A and B, *P < .05 vs the sham‐operated mice; ^#^ *P* < .05 vs the CLP‐induced septic mice treated with lentivirus harboring oe‐NC. In panel C, *P < .05 vs the RAW264.7 cells treated with sh‐NC. In panels D‐K, *P < .05 vs the CLP‐induced septic mice treated with lentivirus harboring sh‐NC; ^#^ *P* < .05 vs the CLP‐induced septic mice treated with lentivirus harboring oe‐KLF4. N = 10. Data were expressed as mean ± standard deviation. Data among multiple groups were compared by one‐way ANOVA, followed by Tukey's post hoc test. The experiments were conducted 3 times independently

The focus was then shifted to the expression pattern of ITGA2B in CLP‐induced septic mice. Results of RT‐qPCR and Western blot analysis showed that ITGA2B expression significantly increased in the liver and lung tissues of CLP‐induced septic mice, although the treatment of oe‐KLF4 down‐regulated the levels of ITGA2B (Figure 2A,B). As shown in Figure 2C, sh‐ITGA2B‐1 or sh‐ITGA2B‐2 both significantly knocked‐down ITGA2B expression. After overexpressing KLF4 or the knock‐down of ITGA2B, the survival rate of CLP‐induced septic mice was significantly increased to more than 50%, while subsequent treatment to impose ITGA2B overexpression reversed the benefits of KLF4 overexpression on the survival rate (Figure 2D). The protein levels of ITGA2B were significantly down‐regulated by overexpressing KLF4 or reducing ITGA2B levels. Meanwhile, the effects of KLF4 on the expression of ITGA2B were counteracted by overexpressing ITGA2B (Figure 2E). The expression levels of TNF‐α, IL‐1β and IL‐6 in mouse serum, liver tissues and lung tissues were diminished upon KLF4 overexpression or ITGA2B knock‐down (Figure 2F‐H). Due to the overexpression of KLF4 or down‐regulation of ITGA2B, the interstitial oedema, inflammatory cell infiltration and damage in the liver and lung tissues were alleviated (Figure 2I,J), and the number of bacterial colonies in the ascitic fluid and serum in CLP‐induced septic mice was smaller (Figure 2K). However, when KLF4 and ITGA2B were overexpressed at the same time, the above‐mentioned effects induced by overexpressed KLF4 alone were effectively reversed. To conclude, sepsis in mice could be alleviated by KLF4 through down‐regulation of ITGA2B.

### 3.3 KLF4 overexpression alleviated LPS‐induced inflammatory response by down‐regulating ITGA2B in RAW264.7 cells

**Figure 3:**
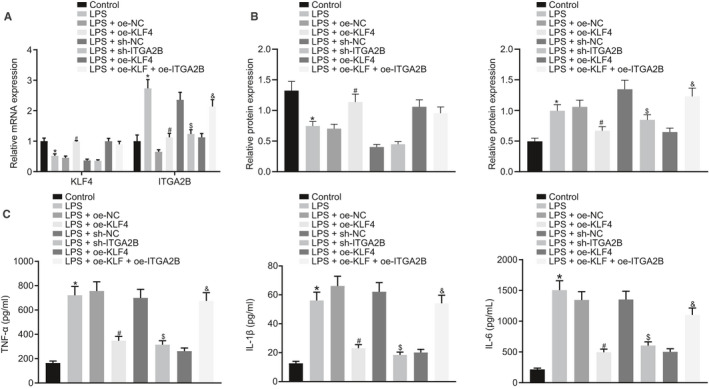
KLF4 overexpression alleviated lipopolysaccharide (LPS)‐induced inflammatory response by down‐regulating ITGA2B in RAW264.7 cells. A, The mRNA expression of KLF4 and ITGA2B in RAW264.7 cells determined by RT‐qPCR. B, The protein expression of KLF4 and ITGA2B in RAW264.7 cells determined by Western blot analysis, normalized to GAPDH. C, The levels of TNF‐α, IL‐1β and IL‐6 in RAW264.7 cell culture medium supernatant determined by ELISA. *P < .05 vs the RAW264.7 cells without any treatment. ^#^ *P* < .05 vs the RAW264.7 cells treated with LPS + oe‐NC. ^$^ *P* < .05 vs the RAW264.7 cells treated with LPS + sh‐NC. ^&^ *P* < .05 vs the RAW264.7 cells treated with LPS + oe‐KLF4. n = 10. Data were expressed as mean ± standard deviation. Data between two groups were compared by independent‐sample *t* test. The experiments were conducted 3 times independently

Next, the role of KLF4 in inflammatory response was explored in LPS‐induced RAW264.7 cells. The expression of KLF4 and ITGA2B was measured by RT‐qPCR and Western blot analysis, which showed that after the induction using LPS, KLF4 expression was reduced, while ITGA2B expression was elevated (Figure 3A,B). According to ELISA analysis, the higher levels of TNF‐α, IL‐1β and IL‐6 induced by LPS tended to decline once KLF4 was overexpressed or when ITGA2B was knocked down (Figure 3C). What is more, the co‐treatment of oe‐KLF4 and oe‐ ITGA2B efficiently neutralized the effects of overexpressing KLF4 alone. These findings revealed that LPS‐induced inflammatory response could be alleviated by KLF4 via inhibition of ITGA2B.

### 3.4 TLR4 promotion of phosphorylation of ERK1/2 negated the inhibitory effects of KLF4 on ITGA2B in RAW264.7 cells

**Figure 4:**
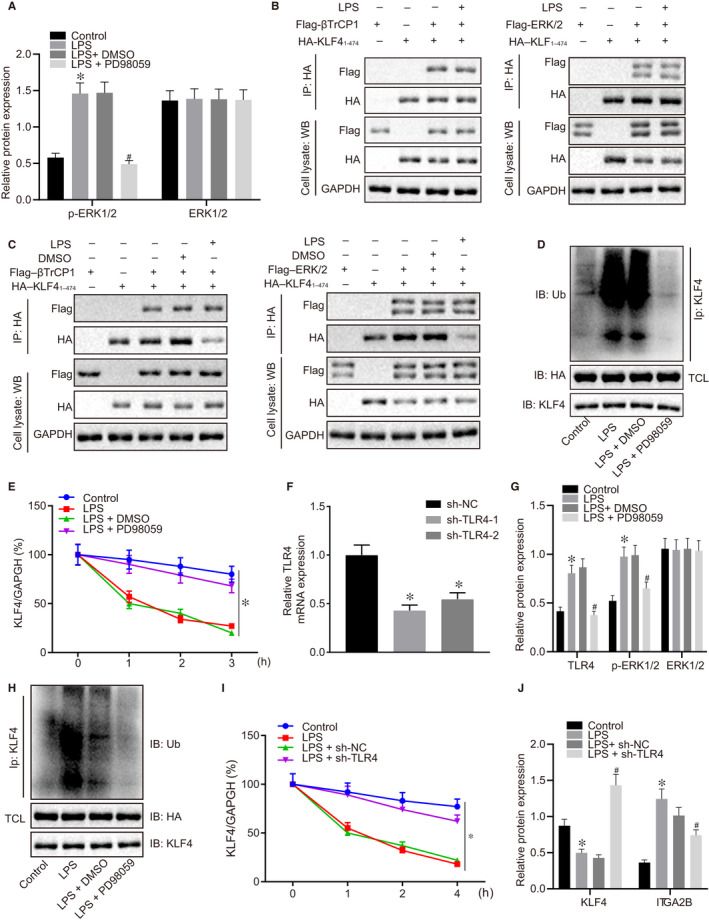
TLR4 promotion of phosphorylation of ERK1/2 negated the inhibitory effects of KLF4 on ITGA2B in RAW264.7 cells. A, The protein expression of ERK1/2 and the extent of ERK1/2 phosphorylation in RAW264.7 cells determined by Western blot analysis, normalized to GAPDH. B, The binding between βTrCP1 or ERK1/2 and KLF4 in RAW264.7 cells after induction of LPS detected by IP assay. C, The binding between βTrCP1 or ERK1/2 and KLF4 in RAW264.7 cells after addition of PD98059 detected by IP assay. D, The deubiquitination of KLF4 in RAW264.7 cells in the presence of PD98059 detected by Western blot analysis. E, The KLF4 protein stability in RAW264.7 cells in the presence of PD98059 and CHX. F, The silencing efficacy of TLR4 in RAW264.7 cells determined by RT‐qPCR. G, The protein expression of TLR4 and ERK1/2 and the extent of ERK1/2 phosphorylation in RAW264.7 cells determined by Western blot analysis, normalized to GAPDH. H, The deubiquitination of KLF4 in RAW264.7 cells when TLR4 is silenced detected by Western blot analysis. I, The KLF4 protein stability in RAW264.7 cells when TLR4 is silenced in the presence of CHX. J, The protein expression of KLF4 and ITGA2B in RAW264.7 cells when TLR4 is silenced determined by Western blot analysis, normalized to GAPDH. In panels E and I, *P < .05 vs the RAW264.7 cells treated with LPS + DMSO. In panel F, *P < .05 vs the RAW264.7 cells treated with LPS + sh‐NC. In panel G, *P < .05 vs the RAW264.7 cells without any treatment; ^#^ *P* < .05 vs the RAW264.7 cells treated with LPS + DMSO. Data were expressed as mean ± standard deviation. Data among multiple groups were compared by one‐way ANOVA, followed by Tukey's post hoc test. The experiments were conducted 3 times independently

According to results of Western blot analysis, the induction of LPS significantly elevated the extent of ERK1/2 phosphorylation, but the protein expression of p‐ERK1/2 declined following the addition of the pharmacological inhibitor (PD98059) (Figure 4A). Next, results of IP assay showed that KLF4 could interact with β‐transducin repeat‐containing protein 1 (βTrCP1) and ERK1/2 in RAW264.7 cells, while the interaction was promoted by LPS induction and weakened by PD98059 treatment (Figure 4B,C). Additionally, the addition of PD98059 to the medium suppressed KLF4 ubiquitination (Figure 4D), and inhibited KLF4 degradation, such that the protein levels of KLF4 were elevated (Figure 4E). Then, the silencing efficacy of sh‐TLR4‐1 and sh‐TLR4‐2 in RAW264.7 cells was evaluated by RT‐qPCR (Figure 4F); due to its higher silencing efficiency, we selected sh‐TLR4‐1 for subsequent experiments. After induction using LPS, protein levels of TLR4 and the extent of ERK1/2 phosphorylation significantly increased, which were all decreased when TLR4 expression was inhibited (Figure 4G). Besides, the inhibition of TLR4 in RAW264.7 cells resulted in suppressed ubiquitination (Figure 4H) and degradation of KLF4, as well as lower protein level of ITGA2B (Figure 4I,J). These findings provide consistent evidence of a central role of the TLR4/ERK1/2/KLF4/ITGA2B axis in sepsis.

### 3.5 The TLR4/ERK1/2/KLF4/ITGA2B axis mediated LPS‐induced inflammatory response in RAW264.7 cells

**Figure 5:**
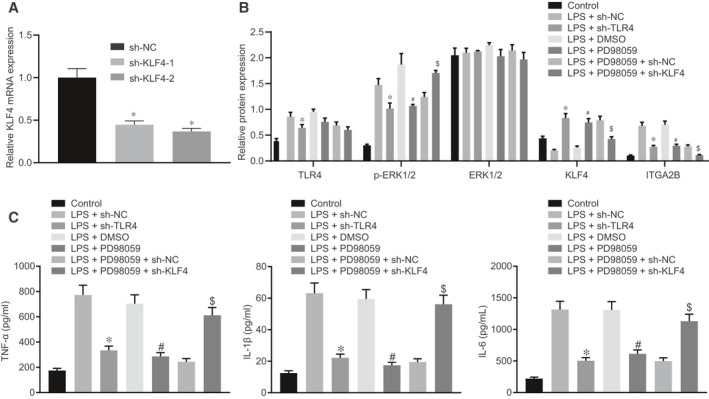
TLR4 aggravated LPS‐induced inflammatory response by suppressing the inhibition of KLF4 on ITGA2B through promoting ERK1/2 phosphorylation. A, The mRNA expression of KLF4 in RAW264.7 cells determined by RT‐qPCR. B, The protein expression of TLR4, ERK1/2, KLF4 and ITGA2B and the extent of ERK1/2 phosphorylation in RAW264.7 cells determined by Western blot analysis, normalized to GAPDH. C, The levels of TNF‐α, IL‐1β and IL‐6 in RAW264.7 cell culture medium supernatant determined by ELISA. *P < .05 vs the RAW264.7 cells treated with LPS + sh‐NC. ^#^ *P* < .05 vs the RAW264.7 cells treated with LPS + DMSO. ^&^ *P* < .05 vs the RAW264.7 cells treated with LPS + PD98059 + sh‐NC. Data were expressed as mean ± standard deviation. Data among multiple groups were compared by one‐way ANOVA, followed by Tukey's post hoc test. The experiments were conducted 3 times independently

Accordingly, we investigated further the role of the TLR4/ERK1/2/KLF4/ITGA2B axis in sepsis. RT‐qPCR results showed that sh‐KLF4‐1 and sh‐KLF4‐2 both significantly knocked‐down KLF4 (Figure 5A). As measured by Western blot analysis, in response to TLR4 knock‐down or PD98059 treatment, protein expression of ITGA2B and extent of ERK1/2 phosphorylation were reduced while KLF4 protein expression was elevated (Figure 5B). Under the same conditions, ELISA indicated reduced levels of TNF‐α, IL‐1β and IL‐6 (Figure 5C). However, the combination of si‐KLF4 and PD98059 reversed these effects induced by either silenced KLF4 or PD98059 treatment alone (Figure 5B,C). Thus, TLR4 promoted the phosphorylation of ERK1/2 to suppress the inhibitory effects of KLF4 on ITGA2B, thereby aggravating LPS‐induced inflammatory response.

### 3.6 The TLR4/ERK1/2/KLF4/ITGA2B axis mediated inflammatory response in CLP‐induced septic mice

**Figure 6:**
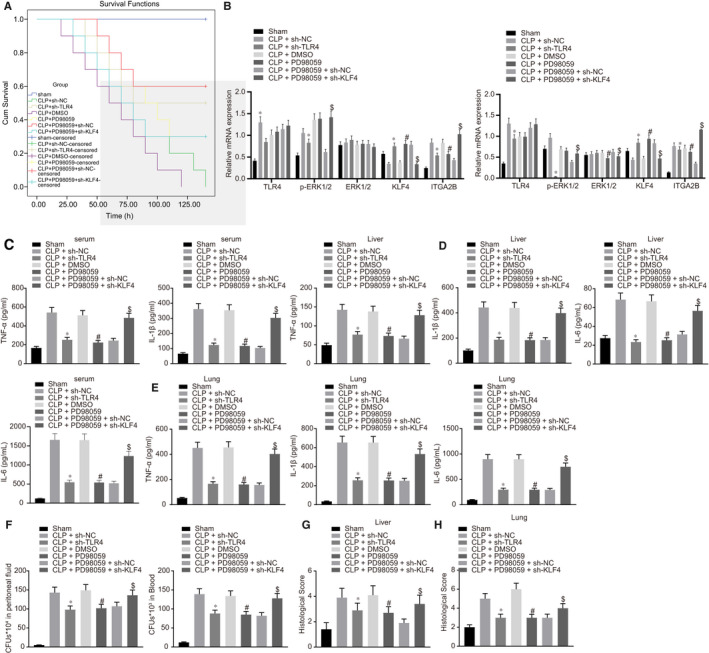
TLR4 promoted sepsis progression in mice by suppressing the inhibitory effects of KLF4 on ITGA2B through promoting ERK1/2 phosphorylation. A, The survival rate of mice at 140 h after caecal ligation and puncture (CLP) surgery. B, The protein expression of TLR4, ERK1/2, KLF4 and ITGA2B and the extent of ERK1/2 phosphorylation in mouse liver and lung tissues at 48 h after CLP surgery determined by Western blot analysis, normalized to GAPDH. C, The serum levels of TNF‐α, IL‐1β and IL‐6 at 48 h after CLP surgery measured by ELISA. D, The levels of TNF‐α, IL‐1β and IL‐6 in mouse liver tissues at 48 h after CLP surgery detected by ELISA. E, The levels of TNF‐α, IL‐1β and IL‐6 in mouse lung tissues at 48 h after CLP surgery determined by ELISA. F, The number of bacterial colonies in mouse PLF and serum. G, Morphological observation and score of liver tissues at 48 h after CLP surgery identified by Haematoxylin‐eosin (HE) staining (200×). H, Morphological observation and score of lung tissues at 48 h after CLP identified by HE staining (200×). *P < .05 vs the CLP‐induced septic mice with lentivirus harboring sh‐NC; ^#^ *P* < .05 vs the CLP‐induced septic mice treated with DMSO; ^$^ *P* < .05 vs the CLP‐induced septic mice treated with PD98059 and lentivirus harboring sh‐NC. n = 10. Data were expressed as mean ± standard deviation. Data among multiple groups were compared by one‐way ANOVA, followed by Tukey's post hoc test. The experiments were conducted 3 times independently

As illustrated in Figure 6A, the survival rate of mice was improved following CLP surgery in mice with TLR4 knock‐down or PD98059 treatment. Further, TLR4 knock‐down or PD98059 treatment resulted in reduced protein expression of ITGA2B, a reduced extent of ERK1/2 phosphorylation, but elevated KLF4 protein expression (Figure 6B). Furthermore, TLR4 knock‐down or PD98059 treatment triggered lower levels of TNF‐α, IL‐1β and IL‐6 in the serum and in liver and lung tissues (Figure 6C‐E), reduced the number of bacterial colonies in the ascitic fluid and serum (Figure 6F), and ameliorated interstitial oedema, inflammatory cell infiltration, and damage in the liver and lung tissues in CLP‐induced septic mice (Figure 6G,H). As expected, KLF4 knock‐down negated and the effects of PD98059 on septic mice. Thus, these findings suggest that the TLR4/ERK1/2/KLF4/ITGA2B axis mediated inflammatory response in CLP‐induced septic mice.

## 4 DISCUSSION

It is estimated that more than one million deaths occur annually in China due to sepsis, with a higher burden of mortality associated with male sex, ageing and comorbidity, together posing a great burden to the public.^20^ Treatments addressing the immunomodulatory mechanisms underlying sepsis are believed to improve the clinical outcomes of patients over a long period of time.^5^ During the current investigation, we aimed to prove the hypothesis that the TLR4/ERK1/2/KLF4/ITGA2B axis is capable of mediating inflammatory response of sepsis.

A primary finding of our study was that KLF4 was down‐regulated in LPS‐induced RAW264.7 cells as well as in the liver and lung tissues of CLP‐induced septic mice, accompanied by activated release of pro‐inflammatory cytokines (TNF‐α, IL‐1β and IL‐6) and unfavourable histological changes. Lung injury is known to occur frequently as a secondary clinical event to sepsis and can be responsible for higher mortality and morbidity.^21^ In addition, liver injury either before or after sepsis is recognized to be of great significance, such that any rescue of liver injury and restoration of liver function might help to reduce mortality and morbidity.^22^ KLF4 is a crucial mediator in the biology of neutrophils, which are key players in innate immune response, as well as a regulator of pro‐inflammatory signalling in macrophages.^23^, ^24^ The production of TNF‐α has been demonstrated to be indicative of sepsis in mice following LPS administration and in HL‐1 cells following LPS treatment.^25^ Also, the reduction in abundance of IL‐1β has been observed to play a role in the suppression of inflammation associated with sepsis in mice.^26^ As a promising biomarker for sepsis, higher IL‐6 concentration correlates with the severity of sepsis.^27^, ^28^ LPS stimulation yielded similar results in bone marrow‐derived macrophages and murine RAW264.7 cells, provoking inhibited KLF4 expression accompanied by increased levels of pro‐inflammatory cytokines (TNF‐α, IL‐1β and IL‐6), while opposite results are seen when KLF4 is overexpressed by treatment with pGMLV‐KLF4.^8^ Consistent with that report, we found that transduction of lentivirus harboring overexpressed KLF4 in mice or delivery of overexpressed KLF4 in RAW264.7 cells tended to block the effects of CLP surgery in vivo or LPS in vitro.

Further mechanistic investigations showed that the action of KLF4 depended on the down‐regulation of highly expressed ITGA2B. The expression of ITGA2B was elevated approximately ninefold in platelets from patients with sepsis, but this aberrant expression resolves in patients who survive sepsis.^10^ Moreover, LPS induction and CLP surgery significantly promoted TLR4 expression and the extent of ERK1/2 phosphorylation. Additionally, the inhibitory effects of KLF4 on ITGA2B were revealed to be repressed by TLR4 through promotion of the extent of ERK1/2 phosphorylation. TLR4 signalling plays an important part in autophagy of RAW264.7 cells induced by LPS, the effects of which are abrogated by TLR4 knock‐down.^29^ Notably, TLR4 activity has the potential to exert suppressive effects on immune dysfunction in sepsis.^30^ Down‐regulation of TLR4 has been proposed as an indicator for amelioration of lung injury in CLP‐induced septic mice after treatment with hesperidin.^31^ Besides, TLR4 knock‐down has been shown to inhibit the expression of KLF4 in human vascular smooth muscle cells.^32^ In neutrophils, deficient KLF4 has been documented to impair the TLR4 signalling, thus underscoring the significance of KLF4/TLR4 in inflammatory reactions.^23^ Following induction of sepsis, the extent of ERK1/2 phosphorylation has been found to be elevated, but declined upon alleviation of liver injury.^12^ Under inflammatory conditions, blocking TLR4 in myeloid‐derived suppressor cells have been verified to diminish the phosphorylation of ERK1/2,^13^ thus supporting present results.

**Figure 7:**
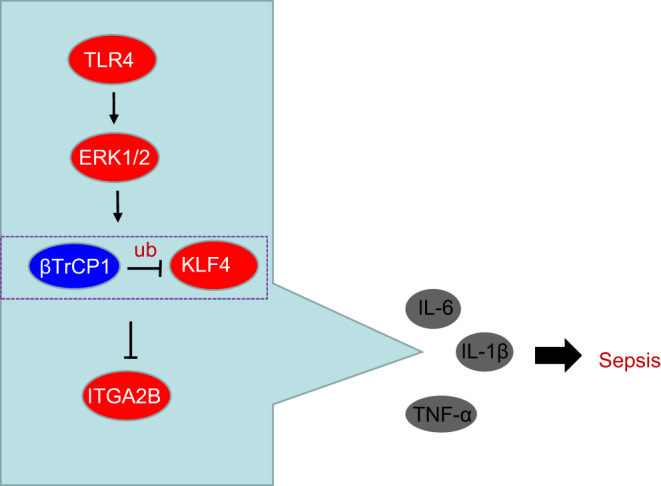
The mechanism diagram depicting that TLR4 activates ERK1/2 to promote the ubiquitination of βTrCP1 on KLF4, which down‐regulates the expression of KLF4. The down‐regulated KLF4 promotes expression of ITGA2B, thereby increasing expression of TNF‐α,IL‐1β and IL‐6, which finally aggravates sepsis. Ub, Ubiquitination

Collectively, the results in our investigation shed light on the anti‐inflammatory role of KLF4 in CLP‐induced septic mice and LPS‐induced RAW264.7 cells, and give insight into the potential mechanism of these effects (Figure 7). KLF4 down‐regulation resulting from promotion by TLR4 of ERK1/2 phosphorylation leads to elevated ITGA2B expression, which underpins the inflammatory response in sepsis. Nevertheless, additional efforts are warranted to determine the translational potential of these findings into the clinical setting.

## CONFLICT OF INTEREST

The authors declare no conflicts of interest.

## AUTHOR CONTRIBUTIONS

Chunwen Li: Conceptualization (equal); Investigation (equal). Lei Yu: Data curation (equal); Writing‐original draft (equal). Chao Mai: Formal analysis (equal); Software (equal). Tianyi Mu: Validation (equal); Writing‐review & editing (equal). Yong Zeng: Methodology (equal); Resources (equal); Supervision (equal).

## Data Availability

Research data are not shared.
